# Onychomycosis Caused by *Fusarium* Species

**DOI:** 10.3390/jof8040360

**Published:** 2022-03-31

**Authors:** Eduardo Vinicius Grego Uemura, Marcelo dos Santos Barbosa, Simone Simionatto, Ahmed Al-Harrasi, Abdullah M. S. Al-Hatmi, Luana Rossato

**Affiliations:** 1 Faculdade de Ciências da Saúde, Universidade Federal da Grande Dourados, Dourados 79862-000, MS, Brazil; eduardouemuraufgd@gmail.com (E.V.G.U.); marcelo_medvet@outlook.com (M.d.S.B.); simonesimionatto@ufgd.edu.br (S.S.); 2 Laboratório de Pesquisa em Ciências da Saúde, Universidade Federal da Grande Dourados, Dourados 79862-000, MS, Brazil; 3 Natural & Medical Sciences Research Center, University of Nizwa, Nizwa 616, Oman; aharrasi@unizwa.edu.om (A.A.-H.); a.alhatmi@unizwa.edu.om (A.M.S.A.-H.); 4 Centre of Expertise in Mycology, Radboud University Medical Centre/Canisius Wilhelmina Hospital, 6532 SZ Nijmegen, The Netherlands

**Keywords:** *Fusarium*, onychomycosis, clinical features, epidemiology, mycology

## Abstract

Onychomycosis is a nail fungal infection that produces nail discolouration, thickness, and separation from the nail bed. The species of the *Fusarium* genus that cause onychomycosis are emerging and the number of cases has increased throughout the years. Microscopic examination, as well as cultures, are required for the accurate diagnosis of onychomycosis. The goal of treatment is to eliminate the organism that causes the disease and restore the nail’s normal appearance. Here, we provide an overview of the onychomycosis cases that have been reported in literature over the last 24 years, which have been caused by the *Fusarium* species. We performed a review on the onychomycosis cases caused by the *Fusarium* species from January 1997 to January 2021. Patients aged between 40 and 49 years made up 30.23% of the cases. The most common aetiologic species was *Fusarium solani* species complex (FSSC), which accounted for 44.11% of the cases, followed by *F. fujikuroi* species complex (FFSC), which accounted for 17.64%; 14.70% of the cases were due to *F. dimerum* species complex (FDSC) and 14.70% of the cases were due *F. oxysporum* species complex (FOSC). Europe accounted for 29.06% of the cases caused by FOSC, whereas Africa accounted for 46.67% of the cases due to FSSC. The clinical presentation of onychomycosis due to *Fusarium* spp. is commonly the distal–lateral pattern of onychomycosis. Identification of the infectious agent in onychomycosis cases due to *Fusarium* is crucial in deciding the proper treatment. Although antifungal susceptibility tests have only been performed in a few cases, susceptibility testing can help with patient management.

## 1. Introduction

As some of the most common infections worldwide, superficial mycoses are becoming a major public health concern [1]. In some cases, superficial fungal infections can progress to invasive infections, which are becoming more common in at-risk populations [2]. Dermatophytes (*tinea unguium*), non-dermatophyte moulds, and yeasts can all cause onychomycosis [3]. Dermatophytes, particularly *Trichophyton mentagrophytes* and *Trichophyton rubrum*, cause approximately 90% of toenail and 75% of fingernail onychomycosis [4]. Non-dermatophyte moulds (NDMs) are an ecologically varied group of fungi that have major habitats such as saprotrophs and plant pathogens, but they are also involved in the aetiology of onychomycosis [5]. The *Fusarium* species, *Aspergillus* species, *Scopulariopsis brevicaulis*, *Neoscytalidium dimidiatum*, and *Acremonium* species are the most commonly isolated NDMs from clinics and hospitals around the world. NDM agents are thought to be responsible for 2–25% of all onychomycosis cases [5]. *Fusarium* species have been implicated as causative agents of opportunistic infections in both humans and animals. Local or systemic predisposing factors frequently trigger human infections, and disseminated illness is linked to weakened immune responses [6]. Onychomycosis caused by the *Fusarium* spp. almost always affects the big toenails, particularly those with traumatic and dystrophic abnormalities, as well as nails infected with dermatophytes [7]. Although early management of mycotic nails in immunocompromised and diabetic patients is critical to avoid life-threatening disease, research on the treatment of onychomycosis caused by the *Fusarium* species is sparse. Onychomycosis caused by the *Fusarium* spp. is little understood in terms of prevalence, clinical symptoms, and mycological features. The clinical, mycological, and epidemiological aspects of onychomycosis caused by *Fusarium* spp. are examined in this review.

## 2. Materials and Methods

The authors used the PubMed database to conduct literature review to find all human clinical episodes of onychomycosis caused by the *Fusarium* spp. Case reports and case series published in the last 24 years, from January 1997 to January 2021, were included in the search approach. The search was limited to English-language literature. The following keywords and concepts were used in conjunction with the “AND” operator: “Onychomycosis and *Fusarium*,” “non-dermatophyte moulds and *Fusarium*,” and “case reports,” “case series,” and “clinical cases.” In addition, all pertinent references referenced in the case reports and reviews were manually searched to uncover other articles not found in the database search. We only included studies in this review that used repeated culture isolations and at least three of the following criteria to identify the *Fusarium* spp. as the sole pathogen causing toenail onychomycosis at the baseline: direct microscopic examination (DME), fungal culture, excluding the dermatophytes, histology, and polymerase chain reaction (PCR) testing. This review only included the *Fusarium* onychomycosis. Non-*Fusarium* species NDMs were excluded, as were NDMs that did not meet our inclusion criteria. First author, year of publication, country, age, gender, aetiologic agents, underlying diseases and risk factors, methods of diagnosis, clinical type, result, treatment, and minimum inhibitory concentration (MIC) values were retrieved from every connected study. We employed the same criteria stated to define the result—mycological, clinical, and cure [5]. Negative KOH microscopy and negative fungal culture were used to determine mycological cure; clinical cure was defined as the development of a completely normal-looking nail; and cure was defined as achieving both the mycological and clinical cure.

## 3. Results

**Figure 1:**
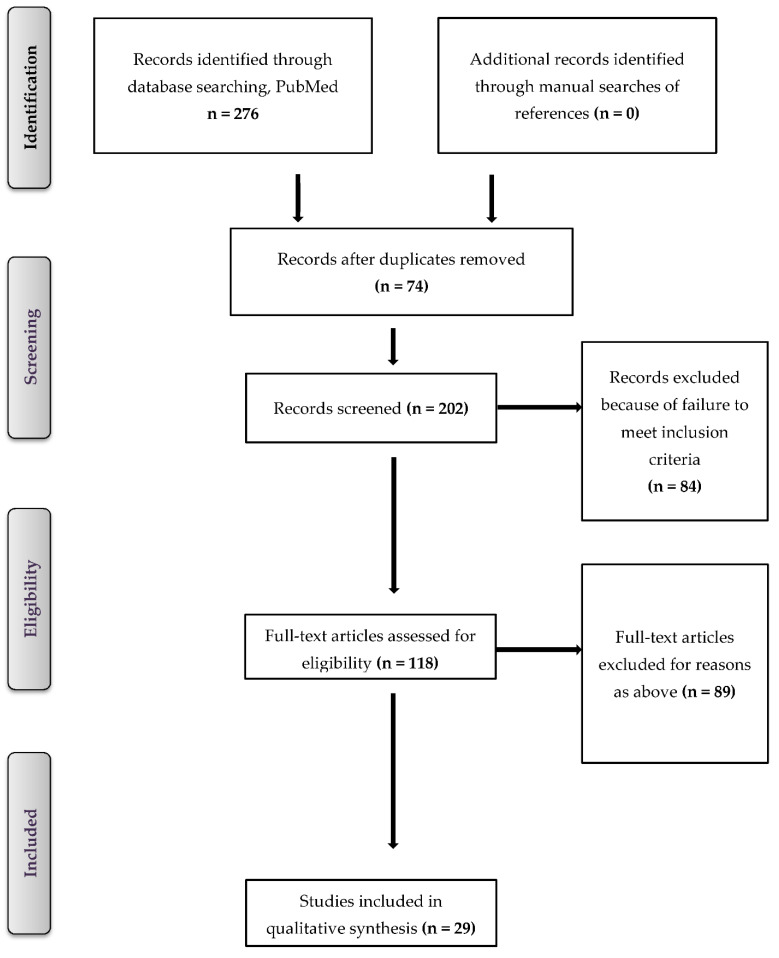
Flow chart presenting the methodology used to obtain the selected articles.

There were 29 studies documenting the *Fusarium* spp. onychomycosis that met our criteria (Figure 1), representing 86 clinical cases from 16 countries (Table 1 and Supplementary Material Table S1). Patients aged 40 to 49 years accounted for 30.23% (26/86) of the 86 cases. The majority of the cases, 66.27% (57/86), included women (Table 1). Table 1 lists the clinical and epidemiological characteristics.

### 3.1. Geographic Distribution of Fusarium Onychomycosis

Table 2 shows the geographic distribution of *Fusarium* onychomycosis. The bulk of the *Fusarium* onychomycosis cases were in Asia, accounting for 39.53% (34/86) of all cases. The *Fusarium solani* species complex (FSSC), which accounted for 44.11% (15/34) of the patients, was followed by the *Fusarium fujikuroi* species complex, which accounted for 17.64% (6/34) of the cases. The *Fusarium dimerum* species complex and *Fusarium oxysporum* species complex (FOSC) were both identified in 14.70% (5/34) of the patients. Europe accounted for 29.06% (25/86) of the cases, with FOSC accounting for 52% (13/25) and the *Fusarium* spp. accounting for 28% (7/25) of the total cases. The continent of Africa accounted for 17.44% (15/86), with FSSC accounting for 46.67% (7/15). The *Fusarium* species complex found in 50% (4/8) of the cases (4/8) and FOSC in 50% (4/8) of the cases (4/8) corresponded with 9.30% (8/86) of the descriptions from South America. Only 4.65% (4/86) of the cases in North America were characterised, and the species complex documented was the *Fusarium* spp. in 50% (2/4) and FSSC in 50% (2/4) of the cases, respectively (Table 2).

In Europe, FOSC is the most common *Fusarium* group (ranging from 35% to 56%), followed by the FSSC species (31% to 33% of cases). In the Americas and Asia, however, the FSSC species are the most common *Fusarium* group (65% to 76%), followed by FOSC (24% to 33%), which is consistent with our results (71.43% FSSC and 28.57% FOSC) (Table 3).

### 3.2. Underlying Conditions and Exposure to Risk Conditions

The presence of underlying diseases was recorded in only 16.27% of the *Fusarium* onychomycosis case reports (14/86). Prior to the onset of the fungal infection, 42.8% (6/14) of the cases had a traumatic injury, followed by diabetes mellitus in 28.5% (4/14) of the cases; arterial hypertension in 14.2% (2/14) of the cases; and HIV and autoimmune illness in 7.1% (1/14) of the cases (Table 1).

### 3.3. Site of Infection of Fusarium Onychomycosis

The toenail was the most common site of infection for *Fusarium* onychomycosis, accounting for 46.5% (40/86) of the cases, followed by fingernail (30.23% (26/86)) and fingernail together with toenail (10.46% (09/86)). In 12.79% of the cases (11/86), the location of infection was not specified (Table 1).

### 3.4. Clinical Type and Fusarium Etiological Agent

The most common clinical type of *Fusarium* onychomycosis was distal–⁠lateral subungual onychomycosis (DLSO), which accounted for 41.86% (36/86) of the patients, followed by proximal subungual onychomycosis (PSO), with 16.27% (14/86) of the cases, total dystrophic onychomycosis (TDO), with 13.95% (12/86), and superficial white onychomycosis (SWO), which accounted for 13.95% (12/86) of the cases (Table 3). Furthermore, 1.16% (01/86) of the cases had PSO + WSO, and 20.93% (18/86) of the cases had *Fusarium* onychomycosis with paronychia. The clinical type was not described in 22.09% (19/86) of the 86 clinical cases (Table 3).

In 84.88% (73/86) of the cases, species complex identification (based on colony morphology, microscopic examination, and/or molecular approaches) was possible. Non-identified *Fusarium* species were responsible for just 15.11% (13/86) of the cases. Onychomycosis was caused by the *Fusarium* species complex in 34.88% (30/86) of cases, 30.23% (26/86) by *Fusarium oxysporum* species complex, 13.95% (12/86) by the *Fusarium fujikuroi* species combination, and 5.81% (5/86) by the *Fusarium dimerum* species complex. When comparing the type of infection with the *Fusarium* species complex, we discovered that FOSC predominated in 28.9% (11/38) of the DLSO cases. The major species complex in PSO, TDO, and SWO was the *F. solani* species complex, which was found in 33.33%, 37.50%, and 71.43% of the cases, respectively (Table 3).

### 3.5. Diagnosis

NDMs isolated from onychomycotic nail samples are classified as (i) contaminant, (ii) commensal, (iii) temporary saprobic coloniser, (iv) persistent secondary coloniser, (v) successional invader, and (vi) primary invader. Direct microscopy, fungal culture, histopathology, PCR, flow cytometry, and dermoscopy are some of the diagnostic procedures that have been utilised to diagnose NDMs. The presence of fungal elements such as hyphae, mycelium, and arthrospores can be detected through direct microscopic examination using potassium or sodium hydroxide (KOH or NaOH), sodium sulphide, or Parker’s blue-black permanent ink. In 98.83% (85/86) of the *Fusarium* clinical cases, direct microscopic examination was performed (Table 1).

The vitality of an isolated pathogen is demonstrated by a fungal culture, which verifies the clinical suspicion of onychomycosis. In the laboratory, Sabouraud dextrose agar (SDA) is widely used with antibiotics such as chloramphenicol, with or without cycloheximide added. When NDMs are sown in SDA, they tend to outgrow the slower-growing dermatophytes; however, cycloheximide suppresses the NDM growth while permitting dermatophytes to proliferate. In 100% of the cases (86/86), fungal culture was found (Table 1). Only 36.86% (36/86) of the cases were identified molecularly; DME and culture were also used to identify the cases (Table 1).

### 3.6. Treatment and Outcome

Oral itraconazole was utilised in 26.74% (23/86) of the cases, oral terbinafine in 17.44% (15/86) of the cases, and topical amphotericin B in 8.13% (7/86) of the cases. Thymol solution 3% consists of a colorless crystalline monoterpene phenol that has in vitro antifungal activity, and is considered a potential treatment for onychomycosis. It was used in 8.13% (7/86) of the cases, while nail avulsion was used in 5.81% (5/86), photodynamic therapy was used in 2.32% (2/86), frequent drilling was used in 1.16% (1/86), and diluted clorox soaks were used in 1.16% (1/86) of the cases. A clinical cure was attained in 26.74% of the patients (23/86) and a mycological cure was achieved in 13.95% (12/86) (Supplementary Material Table S1).

### 3.7. Antifungal Susceptibility Test

Table 4 summarises the MIC values of oral and topical antifungal medications against *Fusarium* clinical isolates from onychomycosis. Due to the wide range of susceptibility, it is crucial to identify the causal organism down to the species level to choose the right antifungal therapy.

## 4. Discussion

According to the literature, the prevalence of onychomycosis caused by *Fusarium* spp. in North America is 13.8%, with global prevalence ranging from 2% to 18% [8,9]. *Fusarium falciforme* and *F. keratoplasticum* were the most common *Fusarium* spp. recovered from individuals with onychomycosis in Thailand [10]. The *Fusarium solani* species complex and the FOSC species complex were the most common species in patients with onychomycosis, according to Brazilian research [11]. With immigration, travel across nations, changes in climatic conditions, and demographics, the prevalence and distribution of the *Fusarium* spp. that cause onychomycosis may fluctuate among regions. As a result, clinicians need to be aware of the presence of the common fungal species in their area.

Predisposition to onychomycosis is known to be caused by genetic, environmental, and systemic factors, as well as local nail features. These variables may be related to a poor antifungal treatment response [12]. Risk factors for NDM onychomycosis include climate, age, occlusive footwear, hyperhidrosis, local nail damage, family history, chronic skin illnesses, and occupational exposures. In addition, diabetes, and peripheral vascular disease, as well as immunological suppression, particularly after HIV infection, predispose individuals to *Fusarium* spp.-caused proximal subungual onychomycosis (PSO) [13,14]. In our study, onychomycosis was found as a lesion before dissemination in 259 published cases of invasive fusariosis. As a result, patients with immunodeficiency and onychomycosis caused by the *Fusarium* spp. must be closely monitored [15].

Toenails are more commonly affected by onychomycosis than fingernails due to recurrent trauma, slower growth, a larger nail plate, regular exposure to a damp environment in enclosed footwear, and limited blood flow to the underlying tissues [16,17]. The clinical classification is based on the location and mode of invasion of the nail unit, the clinical pattern of the infection, and histopathological findings. The type of lesion discovered in the nail unit can aid in determining the aetiological agent. Onychomycosis is classified into five types: distal and lateral subungual onychomycosis (DLSO), superficial onychomycosis (SO), proximal subungual onychomycosis (PSO), total dystrophic onychomycosis (TDO), endonyx onychomycosis (EO), and, more recently, mixed pattern onychomycosis (MPO) [18]. The organisms that cause onychomycosis are dermatophytes, non-dermatophyte moulds (NDMs), and yeasts. Most dermatophyte nail infections (60–70%) are caused by *Trichophyton rubrum* and *Trichophyton mentagrophytes* [9]. Several studies have found that the *Fusarium* spp. cause up to 10% of onychomycosis when aetiological agents are classified to the species level [11].

New diagnostic tools, such as molecular biology techniques, are increasingly being utilised. The fungal DNA extracted from clinical samples is amplified by polymerase chain reaction (PCR), which can then be analysed qualitatively and quantitatively using real-time PCR (RT-PCR). By amplification of 28S rDNA, nested PCR has been utilised to detect *Aspergillus* spp., *Fusarium oxysporum*, and *Scopulariopsis brevicaulis* [19,20].

Treatment options for NDM onychomycosis are still restricted and unoptimised, according to the literature; unlike dermatophytes, these fungi do not respond well to systemic antifungal medications [21,22]. However, onychomycosis caused by the *Fusarium* species has not yet been explored. Itraconazole pulse therapy (400 mg per pulse for three pulses; one pulse is 400 mg per day for 1 week, with 3 weeks off therapy) and the other itraconazole pulse therapy regimens have shown modest evidence of clearing *Fusarium* toenail infections [23,24]. *Fusarium* onychomycosis has been successfully treated in Japan using topical efinaconazole, oral itraconazole, and oral fosravuconazole (licenced in Japan since 2018 for *tinea unguium*) [25]. In an onychomycosis patient infected with *Fusarium falciforme* which was resistant to itraconazole and terbinafine, posaconazole pulse therapy (800 mg pulse for four pulses; one pulse is 800 mg/day for 1 week, with 3 weeks off medication) achieved mycological and clinical cure [14]. After performing antifungal susceptibility testing, case studies in the literature have indicated successful treatment of terbinafine- and itraconazole-resistant *Fusarium* species with posaconazole therapy [14]. Polyenes, such as topical amphotericin B, which has a broad antifungal spectrum, have been used successfully in treating a series of *Fusarium* onychomycosis cases in patients who had shown resistance to oral and topical antifungal medications [26]. In recent years, several studies on the susceptibility of dermatophytes, yeasts, and NDMs in vitro have been published in the literature [27,28,29]. To determine the minimum inhibitory concentration (MIC), fungal growth is measured in the presence of various antifungal drug concentrations. This confirms the efficacy of antifungal medications [30,31].

## 5. Conclusions

In conclusion, with a higher prevalence of onychomycosis cases in Asia and Europe, respectively, the *Fusarium* species are emerging pathogens according to this review. Adults, particularly women, are the most afflicted, and the toenail is the most common site of the infection. Although these nail infections are mostly seen in immunocompetent patients, they could possibly serve as a gateway for widespread infections in immunocompromised people. Direct microscopic examination and culture have been used to diagnose fungal infections in most patients. It is critical to identify the infectious agent in onychomycosis at the species level to provide proper treatment. Although this study does not reveal a high mycological or clinical cure rate, it is well recognised that cure is not always attained, even when antifungal medications are adequate for the identified aetiological agent. Although antifungal susceptibility tests have only been performed in a few cases, susceptibility testing can help with treatment management.

## Figures and Tables

**Table 1:** Demographic and clinical characteristics of 86 cases of *Fusarium* onychomycosis.

Variable	N (86)	%	95% CI
Age			
<19	1	1.16	0.2–6.3
20–29	8	9.30	4.79–17.3
30–39	15	17.44	10.86–26.8
40–49	26	30.23	21.54–40.61
50–59	19	22.09	14.62–31.95
60–69	11	12.79	7.29–21.47
>70	5	5.81	2.51–12.29
Not described	1	1.16	0.21–6.3
Gender			
Woman	57	66.28	55.78–75.38
Man	21	24.42	16.56–34.46
Not described	8	9.30	4.79–17.3
Site of infection			
Fingernail	26	30.23	21.54–40.61
Toenail	40	46.51	36.35–56.98
Fingernail and Toenail	9	10.46	5.60–18.71
Not described	11	12.79	7.29–21.47
Fungal Diagnostic			
DME	85	98,83	93.83–99.97
Culture	86	100	95.89–100
PCR	36	41.86	30.54–51.81
DME + Culture + PCR	36	41.86	30.54–51.81
Underlying conditions			
Not described	72	83.72	74.51–90.05
Hypertension	2	2.32	0.64–8.09
Diabetes	4	4,65	1.82–11.36
HIV	1	1.16	0.21–6.30
Autoimmune disease	1	1.16	0.21–6.30
Trauma	6	6.97	3.24–14.40
Outcome			
Mycological cure	12	13.95	7.46–21.00
Clinical Cure	23	26.74	16.89–34.05
Not cured	10	11.62	5.88–18.49
Not described	49	56.97	42.15–61.94

**Table 2:** Prevalence of *Fusarium* onychomycosis by year and geographic location.

Author	Year of Publication	Geographic Region	NDMs Clinical Cases (106) *	*Fusarium* spp. [86 (81.13%)]	*Fusarium* Species Complex
		**Asia**			
Lee et al.	2002	South Korea	1	1 (100%)	*Fusarium solani* species complex
Hattori et al.	2005	Japan	2	2 (100%)	*Fusarium fujikuroi* species complex
Wu et al.	2009	Taiwan	1	1 (100%)	*Fusarium solani* species complex
Yang et al.	2011	South Korea	2	2 (100%)	*Fusarium solani* species complex
Kuruvilla et al.	2012	India	1	1 (100%)	*Fusarium solani* species complex
Ikeda et al.	2014	Japan	1	1 (100%)	*Fusarium oxysporum* species complex
Park et al.	2011	South Korea	1	1 (100%)	*Fusarium oxysporum* species complex
Ranawaka et al.	2012	Sri Lanka	5	2 (40%)	*Fusarium* spp.
Ranawaka et al.	2015	Sri Lanka	5	1 (20%)	*Fusarium dimerum* species complex
Ranawaka et al.	2015	Sri Lanka	8	8 (100%)	*Fusarium oxysporum* species complex; *Fusarium dimerum* species complex; *Fusarium* spp.
Noguchi et al.	2017	Japan	1	1 (100%)	*Fusarium fujikuroi* species complex
Khurana et al.	2018	India	1	1 (100%)	*Fusarium solani* species complex
Gupta et al.	2016	India	11	11 (100%)	*Fusarium fujikuroi* species complex; *Fusarium solani* species complex
Hirose et al.	2020	Japan	1	1 (100%)	*Fusarium oxysporum* species complex
Cases in Asia			**41 (36.68%)**	**34 (39.53%)**	
		**Europe**			
Gianni et al.	1997	Italy	4	4 (100%)	*Fusarium oxysporum* species complex
Baran et al.	1997	Italy	3	3 (100%)	*Fusarium oxysporum* species complex
Vella Zahra et al.	2003	Malta	4	2 (50%)	*Fusarium oxysporum* species complex; *Fusarium solani* species complex
Baran et al.	2004	France	1	1 (100%)	*Fusarium* spp.
Brasch et al.	2011	Germany	1	1 (100%)	*Fusarium fujikuroi* species complex
Brasch et al.	2009	Germany	1	1 (100%)	*Fusarium solani* species complex
Baudraz-Rosselet et al.	2010	Switzerland	8	5 (62.5%)	*Fusarium oxysporum* species complex; *Fusarium fujikuroi* species complex; *Fusarium solani* species complex
Gilaberte et al.	2011	Spain	2	1 (50%)	*Fusarium oxysporum* species complex
Lurati et al.	2011	Switzerland	8	6 (75%)	*Fusarium* spp.
Brasch et al.	2012	Germany	1	1 (100%)	*Fusarium oxysporum* species complex
Cases in Europe			33 (31.13%)	25 (29.07%)	
		**Africa**			
Diongue et al.	2017	Senegal	17	15 (88.2%)	*Fusarium oxysporum* species complex; *Fusarium solani* species complex; *Fusarium fujikuroi* species complex
Cases in Africa			17 (16.04%)	15 (17.44%)	
		**South America**			
Godoy et al.	2004	Brazil	8	8 (100%)	*Fusarium oxysporum* species complex; *Fusarium solani* species complex
Cases in South America			8 (7.55%)	8 (9.30%)	
		**North America**			
Tseng et al.	2000	USA	1	1 (100%)	*Fusarium* spp.
Summerbell et al.	2005	Canada	4	1 (25%)	*Fusarium solani* species complex
Schmidt et al.	2015	USA	1	1 (100%)	*Fusarium* spp.
Al-Hatmi et al.	2015	Mexico	1	1 (100%)	*Fusarium solani* species complex
Cases in North America			7 (6.60%)	4 (4.65%)	
**Total**			**106 (100%)**	**86 (100%)**	

* Considering all cases of *Fusarium* spp. and other NDMs reported in each study.

**Table 3:** Clinical types and corresponding *Fusarium* species complex.

Genera or Species	n (86)	%	Clinical Type													
			DLSO (38)	% (43.18)	PSO (17)	% (20.45)	TDO (8)	% (9.09)	SWO (7)	% (7.95)	DLSO + TDO (3)	% (3.41)	PSO + SWO (1)	% (1.13)	ND (12)	% (13.64)
*F. solani* species complex	30	34.88	11	28.95	6	33.33	2	25.00	5	71.43	1	33.33	0	0.00	5	33.33
*F. oxysporum* species complex	26	30.23	9	23.68	6	33.33	3	37.50	1	14.29	0	0.00	0	0.00	8	58.33
*F. fujikuroi* species complex	12	13.95	9	23.68	3	16.67	0	0.00	0	0.00	0	0.00	0	0,00	1	8.33
*F. dimerum* species complex	5	5.81	3	7.89	0	0.00	0	0.00	0	0.00	2	66.67	0	0.00	0	0.00
Undetermined *Fusarium* spp.	13	15.11	6	15.79	2	16.67	3	37.50	1	14.29	0	0.00	1	1.13	0	0.00

TDO: total dystrophic onychomycosis; PSO: proximal subungual onychomycosis; DLSO: distal and lateral subungual onychomycosis; SWO: superficial white onychomycosis; ND: not described.

**Table 4:** Range of minimum inhibitory concentration (MIC) of common antifungal agents described for *Fusarium* spp. causing onychomycosis (µg/mL).

Study	Species Complex	Terbinafine	Itraconazole	Fluconazole	Voriconazole	Posaconazole	Efinaconazole	Amphotericin B	5-Fluorocytosine	Miconazole
Hirose et al., 2020	FOSC	2	>16	64	2	-	0.5	1	>64	16
Khurana et al., 2018	FSSC	4	32	-	32	32	-	-	-	-
Noguchi et al., 2017	FFSC	>16	>16	>64	2	-	0.13	2	>64	>16
Gupta et al., 2016	FFSC	-	>64	>64	4	1	-	2	>64	-
Gupta et al., 2016	FSSC	-	>64	>64	2	>16	-	2	>64	-
Gupta et al., 2016	FSSC	-	>64	>64	2	>16	-	2	>64	-
Gupta et al., 2016	FSSC	-	>64	>64	2	0.5	-	2	>64	-
Gupta et al., 2016	FFSC	-	>64	>64	4	0.5	-	0.5	>64	-
Gupta et al., 2016	FSSC	-	>64	>64	8	>16	-	2	>64	-
Gupta et al., 2016	FFSC	-	>64	>64	1	0.5	-	2	>64	-
Gupta et al., 2016	FSSC		>64	>64	2	>16	-	2	>64	-
Gupta et al., 2016	FSSC	-	>64	>64	2	>16	-	2	>64	-
Gupta et al., 2016	FSSC	-	>64	>64	1	>16	-	0.5	>64	-
Gupta et al., 2016	FSSC	-	>64	>16	2	>16	-	1	>64	-
Al-Hatmi et al., 2015	FSSC	-	> 16	> 64	8	0.5	-	0.5	-	-
Ikeda et al., 2014	FSSC	>32	>8	>64	2	>16	-	4	>64	-

FFSC: *Fusarium fujikuroi* species complex; FOSC: *Fusarium oxysporum* species complex; FSSC: *Fusarium solani* species complex; -: not described.

## Data Availability

Not applicable.

## Supplementary Materials

The following supporting information can be downloaded at: https://www.mdpi.com/article/10.3390/jof8040360/s1, Table S1: Case reports on the treatment of *Fusarium* spp. Onychomycosis.

## References

[1] Sharma B., Nonzom S. (2021). Superficial mycoses, a matter of concern: Global and Indian scenario-an updated analysis. Mycoses.

[2] Rossato L., Carlesse F., Nobrega de Almeida J., Kontoyiannis D.P., Colombo A.L. (2021). How different is invasive fusariosis in pediatric patients than in adults? A systematic review. Curr. Opin. Infect. Dis..

[3] Hwang S.M., Suh M.K., Ha G.Y. (2012). Onychomycosis due to nondermatophytic molds. Ann. Dermatol..

[4] Gupta A.K., Sibbald R.G., Andriessen A., Belley R., Boroditsky A., Botros M., Chelin R., Gulliver W., Keast D., Raman M. (2015). Toenail Onychomycosis-A Canadian Approach with a New Transungual Treatment: Development of a Clinical Pathway. J. Cutan. Med. Surg..

[5] Gupta A.K., Summerbell R.C., Venkataraman M., Quinlan E.M. (2021). Nondermatophyte mould onychomycosis. J. Eur. Acad. Dermatol. Venereol..

[6] Al-Hatmi A.M., Meis J.F., de Hoog G.S. (2016). *Fusarium*: Molecular Diversity and Intrinsic Drug Resistance. PLoS Pathog..

[7] Baran R., Tosti A., Piraccini B.M. (1997). Uncommon clinical patterns of *Fusarium* nail infection: Report of three cases. Br. J. Dermatol..

[8] Thomas J., Jacobson G.A., Narkowicz C.K., Peterson G.M., Burnet H., Sharpe C. (2010). Toenail onychomycosis: An important global disease burden. J. Clin. Pharm. Ther..

[9] Ghannoum M.A., Hajjeh R.A., Scher R., Konnikov N., Gupta A.K., Summerbell R., Sullivan S., Daniel R., Krusinski P., Fleckman P. (2000). A large-scale North American study of fungal isolates from nails: The frequency of onychomycosis, fungal distribution, and antifungal susceptibility patterns. J. Am. Acad. Dermatol..

[10] van Diepeningen A.D., Feng P., Ahmed S., Sudhadham M., Bunyaratavej S., de Hoog G.S. (2015). Spectrum of *Fusarium* infections in tropical dermatology evidenced by multilocus sequencing typing diagnostics. Mycoses.

[11] Rosa P.D., Heidrich D., Corrêa C., Scroferneker M.L., Vettorato G., Fuentefria A.M., Goldani L.Z. (2017). Genetic diversity and antifungal susceptibility of *Fusarium* isolates in onychomycosis. Mycoses.

[12] Gupta A.K., Konnikov N., Lynde C.W., Summerbell R.C., Albreski D., Baran R., Doncker P.D., Degreef H. (1999). Onychomycosis: Predisposed populations and some predictors of suboptimal response to oral antifungal agents. Eur. J. Dermatol..

[13] Baran R., McLoone N., Hay R.J. (2005). Could proximal white subungual onychomycosis be a complication of systemic spread? The lessons to be learned from Maladie Dermatophytique and other deep infections. Br. J. Dermatol..

[14] Al-Hatmi A.M., Bonifaz A., Calderón L., Curfs-Breuker I., Meis J.F., van Diepeningen A.D., de Hoog G.S. (2015). Proximal subungual onychomycosis caused by *Fusarium falciforme* successfully cured with posaconazole. Br. J. Dermatol..

[15] Nucci M., Varon A.G., Garnica M., Akiti T., Barreiros G., Trope B.M., Nouér S.A. (2013). Increased incidence of invasive fusariosis with cutaneous portal of entry, Brazil. Emerg. Infect. Dis..

[16] Westerberg D.P., Voyack M.J. (2013). Onychomycosis: Current trends in diagnosis and treatment. Am. Fam. Physician.

[17] Gupta A.K., Mays R.R. (2018). The Impact of Onychomycosis on Quality of Life: A Systematic Review of the Available Literature. Skin Appendage Disord..

[18] Hay R.J., Baran R. (2011). Onychomycosis: A proposed revision of the clinical classification. J. Am. Acad. Dermatol..

[19] Ebihara M., Makimura K., Sato K., Abe S., Tsuboi R. (2009). Molecular detection of dermatophytes and nondermatophytes in onychomycosis by nested polymerase chain reaction based on 28S ribosomal RNA gene sequences. Br. J. Dermatol..

[20] Gupta A.K., Nakrieko K.A. (2014). Molecular determination of mixed infections of dermatophytes and nondermatophyte molds in individuals with onychomycosis. J. Am. Podiatr. Med. Assoc..

[21] Tosti A., Piraccini B.M., Lorenzi S. (2000). Onychomycosis caused by nondermatophytic molds: Clinical features and response to treatment of 59 cases. J. Am. Acad. Dermatol..

[22] Gupta A.K., Venkataraman M., Renaud H.J., Summerbell R.C., Shear N.H., Piguet V. (2021). A Paradigm Shift in the Treatment and Management of Onychomycosis. Skin Appendage Disord..

[23] De Doncker P.R., Scher R.K., Baran R.L., Decroix J., Degreef H.J., Roseeuw D.I., Havu V., Rosen T., Gupta A.K., Piérard G.E. (1997). Itraconazole therapy is effective for pedal onychomycosis caused by some nondermatophyte molds and in mixed infection with dermatophytes and molds: A multicenter study with 36 patients. J. Am. Acad. Dermatol..

[24] Gupta A.K., Gregurek-Novak T., Konnikov N., Lynde C.W., Hofstader S., Summerbell R.C. (2001). Itraconazole and terbinafine treatment of some nondermatophyte molds causing onychomycosis of the toes and a review of the literature. J. Cutan. Med. Surg..

[25] Noguchi H., Matsumoto T., Kimura U., Hiruma M., Kano R., Yaguchi T., Ihn H. (2020). Non-dermatophyte Mould Onychomycosis in Japan. Med. Mycol. J..

[26] Lurati M., Baudraz-Rosselet F., Vernez M., Spring P., Bontems O., Fratti M., Monod M. (2011). Efficacious treatment of non-dermatophyte mould onychomycosis with topical amphotericin B. Dermatology..

[27] Jo Siu W.J., Tatsumi Y., Senda H., Pillai R., Nakamura T., Sone D., Fothergill A. (2013). Comparison of in vitro antifungal activities of efinaconazole and currently available antifungal agents against a variety of pathogenic fungi associated with onychomycosis. Antimicrob. Agents Chemother..

[28] Gupta A.K., Paquet M. (2015). Management of Onychomycosis in Canada in 2014. J. Cutan. Med. Surg..

[29] Curatolo R., Juricevic N., Leong C., Bosshard P.P. (2021). Antifungal susceptibility testing of dermatophytes: Development and evaluation of an optimised broth microdilution method. Mycoses.

[30] Sanguinetti M., Posteraro B. (2017). New approaches for antifungal susceptibility testing. Clin. Microbiol. Infect..

[31] Arendrup M.C., Kahlmeter G., Guinea J., Meletiadis J. (2021). How to: Perform antifungal susceptibility testing of microconidia-forming dermatophytes following the new reference EUCAST method E.Def 11.0, exemplified by Trichophyton. Clin. Microbiol. Infect..

