# Supplementary material for: Onychomycosis Caused by Fusarium Species
Source: J Fungi (Basel). 2022 Mar 31;8(4):360. doi: 10.3390/jof8040360 (PMC9027400; doi:10.3390/jof8040360)
Supplement: Supplementary file 1 [file jof-08-00360-s001.zip › jof-1635909-supplementary.pdf]

Journal of
*Fungi*

## Supplementary Material

**Table S1.** Case reports on the treatment of *Fusarium* spp. onychomycosis.

| Study | Microorganism | Clinical Presentation | Paronychia | Antifungal Treatments | Other antifungal treatments | Clinical Cure | Mycological Cure |
|---|---|---|---|---|---|---|---|
| Hirose et al., 2020 | FOSC | PSO | Yes | 10% efinaconazole solution during six months, cure was achieved after 12 months, no recurrence of onychomycosis or paronychia has been detected. | - | Achieved | Achieved |
| Khurana et al., 2018 | FSSC | TDO | No | Treated previously with courses of terbinafine, itraconazole, and griseofulvin. No improvement in the appearance of the nail was observed. | Q-switched Nd-YAG laser | Achieved | Achieved |
| Diongue et al., 2017 | FSSC | DLSO | No | - | - | - | - |
| Diongue et al., 2017 | FSSC | - | No | - | - | - | - |
| Diongue et al., 2017 | FOSC | - | No | - | - | - | - |
| Diongue et al., 2017 | FSSC | DLSO | No | - | - | - | - |
| Diongue et al., 2017 | FOSC | - | No | - | - | - | - |
| Diongue et al., 2017 | FOSC | DLSO | No | - | - | - | |
| Diongue et al., 2017 | FFSC | DLSO | No | - | - | - | - |
| Diongue et al., 2017 | FFSC | DLSO | No | - | - | - | - |
| Diongue et al., 2017 | FSSC | - | No | - | - | - | - |
| Diongue et al., 2017 | FSSC | - | No | - | - | - | - |
| Diongue et al., 2017 | FFSC | - | Yes | - | - | - | - |
| Diongue et al., 2017 | FSSC | - | No | - | - | - | - |
| Diongue et al., 2017 | FSSC | - | No | - | - | - | - |
| Diongue et al., 2017 | FOSC | DLSO | No | - | - | - | - |

| | | | | | | | |
|---|---|---|---|---|---|---|---|
| Diongue et al., 2017 | FFSC | DLSO | No | - | - | - | - |
| Noguchi et al., 2017 | FFSC | DLSO | No | Oral terbinafine for 25.5 months, this treatment did not achieve cure. Then, 3 months of topical application of 10% efinaconazole solution was prescribed. | - | Achieved | Achieved |
| Gupta et al., 2016 | FFSC | PSO | - | - | - | - | - |
| Gupta et al., 2016 | FSSC | PSO | - | - | - | - | - |
| Gupta et al., 2016 | FSSC | PSO | - | - | - | - | - |
| Gupta et al., 2016 | FSSC | PSO | - | - | - | - | - |
| Gupta et al., 2016 | FFSC | DLSO | - | - | - | - | - |
| Gupta et al., 2016 | FSSC | TDO | - | - | - | - | - |
| Gupta et al., 2016 | FFSC | DLSO | - | - | - | - | - |
| Gupta et al., 2016 | FSSC | TDO | - | - | - | - | - |
| Gupta et al., 2016 | FSSC | PSO | - | - | - | - | - |
| Gupta et al., 2016 | FSSC | DLSO | - | - | - | - | - |
| Gupta et al., 2016 | FSSC | PSO | - | - | - | - | - |
| Al-Hatmi et al., 2015 | FSSC | PSO | Yes | Initially, itraconazole 200 mg/day, changed to terbinafine 250 mg/day for 2-5 months, without clinical improvement. After molecular identification and anti-fungal susceptibility test, posaconazole 800 mg/day for 1 week each month for 4 months was started. | - | Achieved | Achieved |
| Ranawaka et al., 2015 | FDSC | DLSO + TDO | Yes | 400 mg/day 7 days/month itraconazole 3 pulses therapy. | Clotrimazole lotion | Not achieved | Not achieved |
| Ranawaka et al., 2015 | FDSC | TDO | No | 500 mg/day 7 days/month terbinafine 2 pulses therapy. | 3% Thymol in spirit | Achieved | Achieved |
| Ranawaka et al., 2015 | FDSC | DLSO + TDO | No | 400 mg/day 7 days/month itraconazole (2 pulses for fingernails and 3 pulses for toenails). | 3% Thymol in spirit | Not achieved | Not achieved |

| | | | | | | | |
|---|---|---|---|---|---|---|---|
| Ranawaka et al., 2015 | FDSC | DLSO | Yes | 400 mg/day 7 days/month itraconazole 2 pulses therapy. | 3% Thymol in spirit | Not achieved | Not achieved |
| Ranawaka et al., 2015 | *Fusarium* spp. | DLSO | Yes | 400 mg/day 7 days/month itraconazole (2 pulses for fingernails and 3 pulses for toenails). | 3% Thymol in spirit | Not achieved | Not achieved |
| Ranawaka et al., 2015 | FOSC | DLSO + TDO | Yes | 400 mg/day 7 days/month itraconazole (2 pulses). | 3% Thymol in spirit | Not achieved | Not achieved |
| Ranawaka et al., 2015 | FOSC | PSO | Yes | 500 mg/day 7 days/month terbinafine (2 pulses for fingernails and 3 pulses for toenails). | 3% Thymol in spirit | - | - |
| Ranawaka et al., 2015 | FOSC | DLSO | Yes | 500 mg/day 7 days/month terbinafine (2 pulses for fingernails and 3 pulses for toenails). | 3% Thymol in spirit | Not achieved | Not achieved |
| Ranawaka et al., 2015 | FDSC | DLSO | No | 400 mg/day 7 days/month itraconazole 3 pulses therapy. | 3% Thymol in spirit | Not achieved | Not achieved |
| Schmidt et al., 2015 | *Fusarium* spp. | PSO + WSO | No | 250 mg/day × 6 weeks terbinafine. After the initial six weeks of therapy, another six weeks of terbinafine was prescribed. The patient had complete clinical resolution of symptoms. | - | Achieved | - |
| Ikeda et al., 2014 | FSSC | - | Yes | Initially, 2% ketoconazole cream for 3 weeks, but no improvement was observed. Then, terbinafine 125 mg/day was prescribed. After antifungal susceptibility test, topical 1% butenafine and 1% terbinafine cream was begun, without improvement. | Occlusive dressing therapy of 0.5% amorolfine cream. The nail plate and proximal nail fold were covered with plastic wrap for 8 h/day after application of amorolfine | Achieved | Achieved |
| Brasch et al., 2012 | FOSC | - | Yes | Initially, itraconazole 200 mg daily for 10 weeks. The second attempt was itraconazole 400 mg/day for 10 weeks. Then, terbinafine 250 mg/day for 6 weeks. Topical antifungal treatment included amphotericin B. | Topical ciclopirox and polihexanide | - | - |
| Kuruvilla et al., 2012 | FSSC | SWO | No | Itraconazole 200 mg/day for 2 months. | - | Not achieved | Not achieved |
| Ranawaka et al., 2012 | *Fusarium* spp. | DLSO + TDO | Yes | - | - | - | - |
| Ranawaka et al., 2012 | *Fusarium* spp. | DLSO | No | - | - | - | - |
| Brasch et al., 2011 | FFSC | DLSO | No | 100 mg/day itraconazole and bifonazole for 6 weeks previously; 100 mg/day itraconazole for another 4 weeks without improvement; Terbinafine cream. | Ciclopirox plus partly nail plate remove; Complete nail surgical avulsion; Ciclopirox. | Achieved | - |
| Gilaberte et al., 2011 | FOSC | DLSO | - | Initially, oral terbinafine and itraconazole without improvement. | Amorolfine and ciclopirox lacquers; | Achieved | Achieved |

| | | | | | | | |
|---|---|---|---|---|---|---|---|
| | | | | | Methylaminolevulinate photodynamic therapy | | |
| Lurati et al., 2011 | *Fusarium* spp. | DLSO | No | Application of an amphotericin B solution once daily to the affected nails and surrounding tissue. 1–3 drops of the solution during 23 months. | Initially, topical treatment with amorolfine nail lacquer during 6 months | Achieved | - |
| Lurati et al., 2011 | *Fusarium* spp. | TDO | No | Initially, terbinafine 250 mg/day during 3 days. Then, application of an amphotericin B solution once daily to the affected nails and surrounding tissue. 1–3 drops of the solution during 12 months. | - | Achieved | - |
| Lurati et al., 2011 | *Fusarium* spp. | PSO | No | Application of an amphotericin B solution once daily to the affected nails and surrounding tissue. 1–3 drops of the solution during 12 months. | Initially, topical treatment with amorolfine nail lacquer during 6 months - 2 cycles | Achieved | - |
| Lurati et al., 2011 | *Fusarium* spp. | TDO | No | Initially, terbinafine 250 mg/day during 3 days. Then, application of an amphotericin B solution once daily to the affected nails and surrounding tissue. 1–3 drops of the solution during 12 months. | Nail surgical avulsion and imidazole cream during 2 years. | Achieved | - |
| Lurati et al., 2011 | *Fusarium* spp. | DLSO | No | Initially, terbinafine 250 mg/day during 3 months. Then, application of an amphotericin B solution once daily to the affected nails and surrounding tissue. 1–3 drops of the solution during 12 months. | - | Achieved | - |
| Lurati et al., 2011 | *Fusarium* spp. | DLSO | No | Initially, terbinafine 250 mg/day during 3 months, treatment repeated 2x. Then, Application of an amphotericin B solution once daily to the affected nails and surrounding tissue. 1–3 drops of the solution during 11 months. | - | Achieved | - |
| Park et al., 2011 | FOSC | - | No | Itraconazole 400 mg/day for 1 week followed by no medication for 3 weeks during 6 months. | - | Not achieved | Achieved |
| Yang et al., 2011 | FSSC | DLSO | No | Itraconazole 200 mg/day for 3 months. | - | Not achieved | - |
| Yang et al., 2011 | FSSC | DLSO | No | Terbinafine 250 mg/day for 2 months. | - | Not achieved | - |
| Baudraz-Rosselet et al., 2010 | FOSC | PSO | No | Initially, itraconazole 200 mg/day 1 week every month during 3 months; Terbinafine 250 mg/day p.o. during 6 months. Then, terbinafine 250 mg/day during 3 months. 6 – 12 months later, itraconazole pulse therapy 200 mg/day 1 week every month during 3 months. | Topical treatment with amorolfine and nail drilling. | - | - |
| Baudraz-Rosselet et al., 2010 | FOSC | DLSO | No | Initially, terbinafine 250 mg/day during 1 week/month, followed by fluconazole 150 mg/week during 3 weeks, for 3 months. Then, terbinafine 250 mg/day during 3 months and, 6–12 months later, itraconazole pulse therapy 200 mg/day 1 week every month during 3 months. | Nail drilling. | - | - |

| | | | | | | | |
|---|---|---|---|---|---|---|---|
| Baudraz-Ros-selet et al., 2010 | FOSC | DLSO | No | Initially, fluconazole 150 mg/week during 4 months; terbinafine 250 mg/day during 1 week per month, followed by fluconazole 150 mg/week during 3 weeks, for 3 months. Then, terbinafine 250 mg/day during 3 months and, 6–12 months later, itraconazole pulse therapy 200 mg/day 1 week every month during 3 months. | Topical treatment with am-orolfine and nail drilling. | - | - |
| Baudraz-Ros-selet et al., 2010 | FFSC | PSO | No | Initially, itraconazole pulse therapy 200 mg/day 1 week every month during 3 months. Then, terbinafine 250 mg/day during 3 months and, 6–12 months later, itraconazole pulse therapy 200 mg/day 1 week every month during 3 months | Topical treatment with am-orolfine. | - | - |
| Baudraz-Ros-selet et al., 2010 | FSSC | DLSO | No | Terbinafine 250 mg/day during 3 months | - | - | - |
| Brasch et al., 2009 | FSSC | DLSO | No | - | - | - | - |
| Wu et al., 2009 | FSSC | DLSO | No | Itraconazole 400 mg/day | - | Not achieved | Not achieved |
| Hattori et al., 2005 | FFSC | DLSO | No | Itraconazole 200 mg/day for 1 week (no medication for 3 weeks) for 6 months. | - | Achieved | Achieved |
| Hattori et al., 2005 | FFSC | DLSO | No | Initially, the patient received terbinafine 125 mg/day for 4 months, without im-provement. Then, itraconazole 100 mg/day was prescribed for 2 months, but a little improvement was observed. Finally, pulse therapy with itraconazole 200 mg/day for 2 weeks (no medication for 2 weeks) was given. The treatment was carried out for 6 months | - | Achieved | Achieved |
| Summerbell et al., 2005 | FSSC | DLSO | - | - | - | - | - |
| Baran et al., 2004 | *Fusarium* spp. | SWO | No | - | - | - | - |
| Godoy et al., 2004 | FSSC | SWO | - | - | - | - | - |
| Godoy et al., 2004 | FSSC | SWO | - | - | - | - | - |
| Godoy et al., 2004 | FSSC | SWO | - | - | - | - | - |
| Godoy et al., 2004 | FSSC | SWO | - | - | - | - | - |
| Godoy et al., 2004 | FOSC | SWO | - | - | - | - | - |
| Godoy et al., 2004 | FOSC | SWO | - | - | - | - | - |

| | | | | | | | |
|---|---|---|---|---|---|---|---|
| Godoy et al., 2004 | FOSC | SWO | - | - | - | - | - |
| Godoy et al., 2004 | FOSC | SWO | - | - | - | - | - |
| Vella Zahra et al., 2003 | FOSC | - | - | - | - | - | - |
| Vella Zahra et al., 2003 | FSSC | - | - | - | - | - | - |
| Lee et al., 2002 | FSSC | - | No | 3 months with 400 mg/day itraconazole for 7 days/months for 2 months. | - | Not achieved | - |
| Tseng et al., 2000 | *Fusarium* spp. | TDO | | Econazole cream twice daily. After the mycological diagnosis, terbinafine cream twice daily for 4 weeks was prescribed. Lastly, fluconazole 100 mg/day was given. | Diluted clorox soaks | Achieved | - |
| Baran et al., 1997 | FOSC | - | Yes | Topical bifonazole | Nail avulsion; Ciclopirox | Achieved | Achieved |
| Baran et al., 1997 | FOSC | PSO | Yes | - | 8% ciclopirox nail lacquer for 8 months and partial nail avulsion | Achieved | Achieved |
| Baran et al., 1997 | FOSC | - | Yes | - | - | - | - |
| Gianni et al., 1997 | FOSC | TDO | Yes | Itraconazole 200 mg/day for 3 months | - | - | - |
| Gianni et al., 1997 | FOSC | DLSO | Yes | Terbinafine 250 mg/day for 3 months | - | Achieved | - |
| Gianni et al., 1997 | FOSC | - | Yes | Itraconazole 200 mg/day for 3 months | - | Achieved | - |
| Gianni et al., 1997 | FOSC | SWO | No | Terbinafine 250 mg/day for 3 months | - | Achieved | - |

DLSO: Distal and lateral subungual onychomycosis; PSO: Proximal subungual onychomycosis; TDO: Total dystrophic onychomycosis; SWO: Superficial white onychomycosis; Mycological cure: negative KOH and negative fungal culture; Clinical cure: appearance of completely normal-looking nail; - : Not described; FDSC: *Fusarium dimerum* species complex; FFSC: *Fusarium fujikuroi* species complex; FOSC: *Fusarium oxysporum* species complex; FSSC: *Fusarium solani* species complex.
